# Effects of continuous bicycle ergometer and step exercises from admission to discharge in a patient with myelodysplastic syndrome undergoing myeloablative conditioning and hematopoietic stem cell transplantation: A case report

**DOI:** 10.1097/MD.0000000000034001

**Published:** 2023-06-16

**Authors:** Makoto Kawanishi, Yukihide Nishimura, Tokio Kinoshita, Takamasa Hashizaki, Yasunori Umemoto, Kazunari Nishiyama, Fumihiro Tajima

**Affiliations:** a Department of Rehabilitation Medicine, Wakayama Medical University, Wakayama, Japan; b Division of Rehabilitation, Wakayama Medical University Hospital, Wakayama, Japan; c Department of Rehabilitation Medicine, Iwate Medical University, Shiwa-gun, Japan.

**Keywords:** allogeneic hematopoietic stem cell transplant, bicycle ergometer exercise, case report, myelodysplastic syndromes, step exercise

## Abstract

In myelodysplastic syndromes (MDS), in addition to disease-related symptoms, many adverse events are associated with anticancer agents, myeloablative conditioning (MAC), and allogeneic hematopoietic stem cell transplantation (allo-HSCT). Isolation and bed rest in a clean room severely limit physical activity, resulting in cardiopulmonary and muscle weakness. In addition, post-transplant patients may experience general fatigue, gastrointestinal symptoms, and infections associated with a weakened immune system, as well as graft-versus-host disease, which causes further decline in physical function and activities of daily living (ADL). Most reports on the rehabilitation of patients with hematopoietic tumors involve interventions before and after chemotherapy or transplantation. However, an important issue is to establish effective and feasible exercise programs in a clean room setting, where activity is severely limited and physical function is most likely to decline.

**Case report:**

This case report describes the treatment progress of a 60-year-old man with MDS and thrombocytopenia scheduled to receive MAC and allo-HSCT, who continued bicycle ergometer and step exercises from admission to discharge. The patient was admitted for allo-HSCT, and on day 4, he started bicycle ergometer and step exercise in a clean room and continued until discharge. As a result, exercise tolerance and lower-extremity muscle strength were maintained at the time of hospital discharge. Furthermore, the patient was able to continue rehabilitation in a restricted environment without adverse events.

**Conclusions:**

The rehabilitation and treatment course of this case may provide valuable information for patients with MDS and thrombocytopenia.

## 1. Introduction

Myelodysplastic syndromes (MDS) are hematopoietic tumors characterized by blood cell loss due to inactivated hematopoiesis and are caused by acquired genetic abnormalities in hematopoietic stem cells with a high risk of transformation into acute myeloid leukemia.^[1,2]^ The Revised International Prognostic Scoring System for MDS is a prognostic tool commonly used to predict the risk of transformation to leukemia and survival and classifies patients into low-risk and high-risk groups. The low-risk group is treated with supportive care to improve anemia. Whereas, the high-risk group has a poor prognosis, and allogeneic hematopoietic stem cell transplantation (allo-HSCT) is the only treatment of choice to prolong survival.^[3]^ In MDS, in addition to disease-related symptoms, many adverse events are associated with anticancer agents, myeloablative conditioning (MAC), and allo-HSCT. They cause cardiopulmonary and muscular weakness due to clean room isolation and bed rest, which severely limits exercise.^[4–6]^ In addition, general malaise, gastrointestinal symptoms, infections associated with decreased immunity, and graft-versus-host disease may occur after transplantation, further declining physical function and activities of daily living (ADL).^[7,8]^ However, most reports on rehabilitation programs for patients with hematopoietic tumors have been conducted before and after chemotherapy or transplantation.

Rehabilitation from admission to discharge is safe for patients with hematopoietic tumors and thrombocytopenia undergoing transplantation; however, exercise tolerance and muscle strength are reduced in these patients.^[9]^ In addition, even when aerobic exercise and muscle strengthening training are being performed, most reports indicate that low-intensity exercises, such as stretching and ADL training, are being performed during the clean room isolation. Establishing an effective and feasible exercise program is important in this environment, in which activity is severely restricted and physical function is most likely to decline. Recently, it was reported that performing forward and lateral step exercises 3 times a week for 12 weeks significantly increased knee extensor strength and thigh muscle mass in older patients compared with a control group.^[10,11]^ Step exercises can be performed in areas where activity is limited as simple mobile equipment is required.

## 2. Case description

The patient was a 60-year-old man who was independent in all ADL before his illness. His medical history included left testicular cancer, for which he had undergone high-level vasectomy 12 years ago. The patient was diagnosed with low-risk MDS due to pancytopenia during a routine urologic visit 1 year ago, and supportive care was initiated. Six months ago, he had progressive pancytopenia and increased blast counts (white blood cell [WBC] 1140/µL, neutrophil count 216/µL, hemoglobin 8.8 g/dL, platelets 28000/µL, blasts 4%) and was on an allo-HSCT waiting list due to transition from low-risk to high-risk. As he had a fever of 39.0°C with a low neutrophil count (<500) for 1 month, he was diagnosed with febrile neutropenia and was urgently admitted to the hematology department of this hospital.

Rehabilitation was initiated on the fourth day of hospitalization. To assess motor function, the 6-minute walking distance (6MWD) by the 6-minute walking test (6MWT) and knee extension strength using a hand-held dynamometer (μ-tas F-1; ANIMA Co., Tokyo, Japan) were measured at the start of rehabilitation, before MAC, and at discharge. The 6MWT assesses exercise tolerance, and a strong correlation between 6MWD and maximal oxygen uptake has been reported.^[12]^ The 6MWT was performed according to the American Thoracic Society guidelines, and the patient was instructed in advance to rest if a break was required during the examination.^[13]^ He walked on a flat 30-m straight corridor for 6 minutes under maximal effort conditions, and the total distance was measured. The 6MWT was performed once at each measurement point. Knee extensor strength was measured in a sitting position with both upper limbs crossed in front of the chest and hip and knee joints flexed at 90°. A sensor pad was applied to the distal portion of the right lower leg, secured with a post and non-stretchable strap, and a 3-second maximal isometric exercise was performed. The test was performed 3 times with a 1-minute break in between, and the maximum value among the 3 values was noted.^[14]^ Aerobic exercise was performed on a mobile bicycle ergometer (915E; Monark Exercise AB, Vansbro, Sweden) for 20 to 30 minutes with a target heart rate set at 60% exercise intensity using the Karvonen method. The step exercise was performed using a 30-cm movable step bench, with 2 sets of 30 repetitions to the left and right in the forward direction and 2 sets of 30 repetitions to the side.

**Figure 1.**
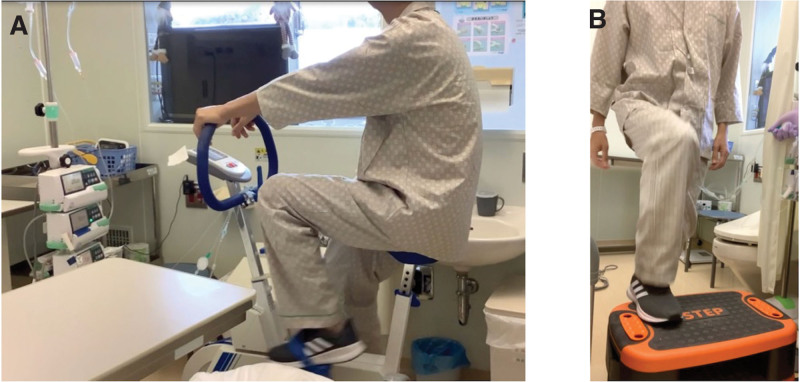
Rehabilitation program in a clean room. (A) Bicycle ergometer exercise in a clean room (hospitalization day 16). (B) Step exercise in a clean room (hospitalization day 16).

Physical examination at the start of rehabilitation revealed anemia in the eyelid conjunctiva but no purpura or petechial hemorrhage in the extremities or trunk. Muscle strength was measured using manual muscle testing according to muscle strength grading.^[15]^ The patient’s manual muscle testing score was grade 5 in all extremities and all ADL were independent; however, his activity level decreased due to anemia and fatigue, and he tended to lie in bed during the day. Laboratory examination revealed WBC 850/µL, hemoglobin 6.3 g/dL, platelet count 11,000/µL, easy infection, anemia, and bleeding due to decreased blood cell counts. At the start of rehabilitation, his 6MWD was 470 m, and knee extension strength was 27.3 kgf on the right and 29.1 kgf on the left. On day 16, the patient was moved to a clean room for MAC and continued with a bicycle ergometer (Fig. 1A) and step exercises (Fig. 1B) indoors. The 6MWD before MAC was 523 m, and knee extension muscle strength was 27.9 kgf on the right and 29.8 kgf on the left. On day 17, MAC therapy with fludarabine and busulfan was initiated (Table 1). On day 21, the patient experienced fatigue and nausea and could no longer sustain the step exercise; therefore, the 30 times, 4 sets were changed to 10 times, 12 sets, and continued. The bicycle ergometer was maintained at the target heart rate. On day 24, allo-HSCT (HLA full-match) was performed. On day 31, acute graft-versus-host disease (skin stage 0, liver stage 0, gastrointestinal stage 2, Grade 3) developed. Owing to diarrhea symptoms and fatigue, the step exercise was changed to 10 times and 12 sets. Laboratory results were WBC 140/µL, neutrophil count 42/µL, hemoglobin 8.0 g/dL, and platelet count 10,000/µL. On day 41, the number of neutrophils increased, and live implantation was confirmed. Laboratory results were WBC 3560/µL, neutrophil count 2990/µL, hemoglobin 8.1 g/dL, and platelet count 34,000/µL. On day 50, the patient was discharged from the clean room. He was able to walk independently in the ward and required no assistance with ADL. On day 59, all ADL were independent, and the patient was discharged. At discharge, the 6MWD was 536 m, and knee extension strength was 29.2 kgf on the right and 30.9 kgf on the left. No serious rehabilitation events occurred between the start of rehabilitation and discharge. The results of the assessment parameters are presented in Table 2, and detailed laboratory results during the study period are presented in Table 3.

**Table 1:** Myeloablative conditioning protocol.

Drugs	Day 17	Day 18	Day 19	Day 20	Day 21	Day 22
Fludarabine (mg)	50	50	50	50	50	50
Busulfan (mg)		205	205	205	205	

**Table 2:** Exercise tolerance and knee extension muscle strength results at each measurement time point.

	At beginning of rehabilitation	Day before MAC	At discharge
Body weight (kg)	65.4	63.2	58.1
6MWD (m)	470	523	536
HHD			
Right (kgf)	27.3	27.9	29.2
Right (kgf/kg)	0.41	0.44	0.50
Left (kgf)	29.1	29.8	30.9
Left (kgf/kg)	0.45	0.47	0.53

HHD = handheld dynamometer, MAC = myeloablative conditioning, 6MWD = 6-minute walk distance.

**Table 3:** Laboratory results at each time point.

	Day 4	Day 18	Day 21	Day 24	Day 25	Day 31	Day 38	Day 41	Day 49	Day 59
WBC (μL)	590	710	460	140	120	150	300	640	2980	2690
Hb (g/dL)	6.2	8.1	8.7	9.2	8.3	8.7	7.8	7.9	8.5	9.9
Plt (μL)	20,000	12,000	33,000	27,000	13,000	17,000	34,000	41,000	191,000	280,000
Neutrophils (μL)			67	94		42	454	2990	1148	1056

Hb = hemoglobin, Plt = platelet, WBC = white blood cell.

Written informed consent was obtained from the patient for the release of this case report. This study complied with all the case report guidelines and reported the required information accordingly.

## 3. Discussion

In previous studies, rehabilitation programs for patients with hematopoietic tumors were conducted safely; however, exercise tolerance and muscle strength were impaired.^[9,16,17]^ Baggena et al showed that step exercise 3 times a week for 12 weeks significantly increased knee extension muscle strength in older adults.^[10,11]^ We noticed that step exercises using a simple, movable step bench can be performed even in a restricted environment. This case report is the first to show that 6MWD and knee extension strength can be maintained in patients with MDS scheduled for MAC and allo-HSCT by continuing rehabilitation, consisting of a bicycle ergometer and step exercises, from admission to discharge. There are few reports of chemotherapy in patients with hematopoietic tumors and rehabilitation before and after transplantation. Wiskemann et al^[17]^ studied patients with hematopoietic tumors who exercised for 20 minutes on a bicycle ergometer and treadmill at an intensity of Borg scale 12 to 14 and resistance training of the upper and lower-extremities with stretch bands at an intensity of Borg scale 14 to 16 three times a week (twice a week under supervision) for 8 to 20 sessions (2–3 sets). As a result, 6MWD decreased from 553 m at admission to 472 m at discharge, and lower-extremity muscle strength decreased from 192.7 N at admission to 168.8 N at discharge. Patients with hematopoietic tumors and scheduled allo-HSCT performed 10 lower-limb muscle strengthening exercises at 60% of maximum lifting capacity, the Karvonen method at 40% bicycle ergometer strength for 15 to 20 minutes, and ADL training 5 times a week for 1 to 4 weeks before transplantation until discharge.^[9]^ They observed a decrease in knee extensor strength from 31.9 to 25.2 kgf and 6MWD from 484.5 to 431.7 m from 3 weeks before transplantation to discharge. In this study, only stretching and manual resistance training were performed during MAC and 2 weeks after allo-HSCT. In another study, hallway walking, stretching, foot treading, and ADL training, were performed for 10 to 20 minutes at 80% load of the watt number measured by the bicycle ergometer test (target HR: 180 – age).^[16]^ The results showed that the maximum watt number was maintained during the lower-extremity ergometer test at discharge, but knee extensor muscle strength decreased from 439 N to 395 N. In a control group (group that performed 10 minutes of exercise and low-intensity training), the muscle strength (decreased from 448 N to 342 N) and the maximum endurance test watt number were significantly reduced. In this report, during clean room isolation, foot stomping and 30-cm step exercises were performed 50 times in 2 sets, but not throughout the entire study period. Our rehabilitation consisted of 20 to 30 minutes of lower-limb ergometer at 60% intensity of the Karvonen method and step exercises using a 30 cm mobile step bench, with 2 sets of 30 times left and right in the forward direction and 2 sets of 30 times in the lateral direction. This was not a higher load or longer duration than in prior studies, but it maintained exercise tolerance and lower-extremity muscle strength. Presumably, the fact that all sessions were conducted under the supervision of a therapist and that the training content remained unchanged during MAC, after allo-HSCT, and clean room management influenced the results, providing an accurate exercise load during the study period.

Patients with thrombocytopenia, especially thrombocytopenia due to disease-related symptoms, chemotherapy, or transplantation, are often restricted to the type and load of exercise due to bleeding risk. Although exercise for patients with hematopoietic tumors has been recommended in Cochrane reviews for improving physical function,^[18]^ specific types and intensities of exercise are not indicated for patients with thrombocytopenia, especially thrombocytopenia. Morishita et al^[19]^ recommended limiting activity when the platelet count is less than 10,000/µL and ADL training such as walking and standing up when the platelet count is between 10,000/µL and 20,000/µL, and that determining the exercise load is primarily subjective and not evidence-based, although most studies have structured their programs according to this criterion. In the present case, the patient had thrombocytopenia due to MDS, and his platelet count was low, ranging from 10,000/µL to <50,000/µL, from the time of admission to positive arrival. However, current loading on the bicycle ergometer and step exercise allowed the patient to continue exercising without adverse events. The present results indicate that the exercise program used in this case can be adapted to many other patients even during thrombocytopenia.

This report is a case report of a single patient. Since patients with hematopoietic tumors have a variety of symptoms and adverse events due to disease, chemotherapy, and transplantation, generalizing the content and course of rehabilitation from admission to discharge is difficult. However, this report showed that the patient was able to perform bicycle ergometer and step exercises continuously from pre-MAC to transplantation and discharge, while maintaining exercise tolerance and lower-extremity muscle strength. The rehabilitation details and course of this case may provide valuable information for patients with MDS who present with thrombocytopenia.

## 4. Conclusions

The bicycle ergometer and step exercises were effective exercise programs for maintaining exercise tolerance and muscle strength because the equipment was easy to move and could be continued from pre-MAC to transplantation and discharge without affecting the inpatient environment. However, for this program to be effective, it is essential that patients continue to exercise during their hospitalization under the supervision of a therapist using precise exercise methods.

## Acknowledgments

The authors thank the clinical nursing staff in the acute care unit for supporting the rehabilitation therapy. The authors also thank Editage (http://www.editage.com) for the English language editing.

## Author contributions

**Conceptualization:** Makoto Kawanishi, Tokio Kinoshita.

**Data curation:** Makoto Kawanishi, Tokio Kinoshita, Takamasa Hashizaki.

**Formal analysis:** Tokio Kinoshita, Yasunori Umemoto.

**Funding acquisition:** Yasunori Umemoto.

**Investigation:** Takamasa Hashizaki.

**Methodology:** Tokio Kinoshita.

**Project administration:** Makoto Kawanishi, Yasunori Umemoto.

**Resources:** Kazunari Nishiyama.

**Software:** Takamasa Hashizaki, Kazunari Nishiyama.

**Supervision:** Yukihide Nishimura, Fumihiro Tajima.

**Validation:** Takamasa Hashizaki.

**Visualization:** Makoto Kawanishi, Kazunari Nishiyama.

**Writing – original draft:** Makoto Kawanishi.

**Writing – review & editing:** Yukihide Nishimura, Tokio Kinoshita, Fumihiro Tajima.
